# Simultaneous Recording of the Uptake and Conversion of Glucose and Choline in Tumors by Deuterium Metabolic Imaging

**DOI:** 10.3390/cancers13164034

**Published:** 2021-08-10

**Authors:** Andor Veltien, Jack van Asten, Niveditha Ravichandran, Robin A. de Graaf, Henk M. De Feyter, Egbert Oosterwijk, Arend Heerschap

**Affiliations:** 1 Department of Medical Imaging (Radiology), Radboud University Medical Centre, 6500HB Nijmegen, The Netherlands; Andor.veltien@radboudumc.nl (A.V.); sjaak.vanasten@radboudumc.nl (J.v.A.); n.ravichandran@science.ru.nl (N.R.); 2 Department of Radiology and Biomedical Imaging, Yale University, New Haven, CT 06520-8043, USA; Robin.degraaf@yale.edu (R.A.d.G.); henk.defeyter@yale.edu (H.M.D.F.); 3 Department of Urology, Radboud University Medical Centre, 6500HB Nijmegen, The Netherlands; Egbert.oosterwijk@radboudumc.nl

**Keywords:** MRI, deuterium metabolic imaging, tumor, ^2^H, glucose, choline

## Abstract

**Simple Summary:**

Tumors increase their glucose and choline uptake to support growth. These properties are employed to detect and identify tumors in the body by imaging the uptake of radio-isotope analogs of these compounds. In this study we show that deuterium metabolic imaging (DMI) (a new MRI method to image metabolites using non-radioactive labeling with deuterium) can image choline uptake in tumors. Furthermore, we demonstrate that DMI can image the tumor uptake of choline and glucose (and additionally its metabolic conversion) simultaneously, in contrast to radio-isotope imaging, which only assesses the uptake of one radio-isotope labeled compound at a time. For these reasons (and also because DMI is relatively simple and can be combined with other MR methods), it is a promising modality for a more specific tumor characterization than by separate imaging of the uptake of radio-isotope labeled glucose or choline.

**Abstract:**

Increased glucose and choline uptake are hallmarks of cancer. We investigated whether the uptake and conversion of [^2^H_9_]choline alone and together with that of [6,6′-^2^H_2_]glucose can be assessed in tumors via deuterium metabolic imaging (DMI) after administering these compounds. Therefore, tumors with human renal carcinoma cells were grown subcutaneously in mice. Isoflurane anesthetized mice were IV infused in the MR magnet for ~20 s with ~0.2 mL solutions containing either [^2^H_9_]choline (0.05 g/kg) alone or together with [6,6′-^2^H_2_]glucose (1.3 g/kg). ^2^H MR was performed on a 11.7T MR system with a home-built ^2^H/^1^H coil using a 90° excitation pulse and 400 ms repetition time. 3D DMI was recorded at high resolution (2 × 2 × 2 mm) in 37 min or at low resolution (3.7 × 3.7 × 3.7 mm) in 2:24 min. Absolute tissue concentrations were calculated assuming natural deuterated water [HOD] = 13.7 mM. Within 5 min after [^2^H_9_]choline infusion, its signal appeared in tumor spectra representing a concentration increase to 0.3–1.2 mM, which then slowly decreased or remained constant over 100 min. In plasma, [^2^H_9_]choline disappeared within 15 min post-infusion, implying that its signal arises from tumor tissue and not from blood. After infusing a mixture of [^2^H_9_]choline and [6,6′-^2^H_2_]glucose, their signals were observed separately in tumor ^2^H spectra. Over time, the [^2^H_9_]choline signal broadened, possibly due to conversion to other choline compounds, [[6,6′-^2^H_2_]glucose] declined, [HOD] increased and a lactate signal appeared, reflecting glycolysis. Metabolic maps of ^2^H compounds, reconstructed from high resolution DMIs, showed their spatial tumor accumulation. As choline infusion and glucose DMI is feasible in patients, their simultaneous detection has clinical potential for tumor characterization.

## 1. Introduction

Enhanced glucose uptake is a hallmark of malignant tumors. For diagnostic purposes, this is traditionally assessed via PET using the glucose analog ^18^Fluordeoxyglucose (FDG) [1]. However, this method cannot monitor subsequent glucose catabolism. For in vivo applications, ^13^C magnetic resonance (MR) spectroscopy has been used to probe uptake and metabolic conversions of non-radioactive ^13^C labeled glucose in tumors into lactate and other compounds [2,3]. The low SNR of the method precludes its use as an imaging method, although recently reported denoising methods may favor better imaging conditions [4]. The hyper-polarization of ^13^C nuclei in labeled compounds considerably improves the signal-to-noise ratio (SNR) of their signals. Applying these compounds to the body enables imaging of metabolic processes otherwise not detectable by MR, but this method is limited by a short time window of visibility. This short time window affects glucose in particular due to short T1 relaxation times of the ^13^C nuclei in this molecule [5,6]. A further possibility for enhanced glucose detection in MR is provided by chemical exchange saturation transfer (CEST) imaging by which proton exchange between water and glucose is employed to exploit the large magnetic pool of water protons for an SNR increase in the detection of the glucose moiety. However, the method may be rather non-specific due to various interactions in which these protons are involved and it also gives no direct view on glucose catabolism [7].

Recently, the use of deuterium (^2^H or D) MR to trace deuterated metabolites in the body (after administration of deuterated substrate) has gained new momentum under the names of deuterium metabolic spectroscopy (DMS) or imaging (DMI), in particular for the spatial monitoring of glucose uptake and its conversion in the brain and brain tumors using non-radioactive [6,6′-^2^H_2_]glucose as substrate [8,9,10]. DMI has several advantages (e.g., by tracking ^2^H in lactate and combined glutamine and glutamate signals, it provides a quantitative biomarker for the Warburg effect [9] and for the glycolytic pathway in tumors [11]). A potential clinical advantage of the method is the simplicity of its application compared to FDG-PET and hyper-polarization MR [9].

Enhanced choline uptake is another hallmark of cancer and the uptake of choline compounds in tumors has been assessed by ^18^F or ^11^C choline PET for diagnostic purposes [12]. Often, choline-PET identifies tumors better than FDG-PET [13,14]. Increased steady state levels of choline compounds in tumors can be mapped by ^1^H MR spectroscopic imaging, but separation of its different species is not possible [12,15]. The main different phosphorylated choline compounds can be detected via ^31^P MR spectroscopic imaging employing ^1^H decoupling or high magnetic fields (≥7T), although at a lower spatial resolution than total choline detection via ^1^H MR spectroscopic imaging [12,16,17]. It is possible to obtain choline in which the nine protons at the methyl groups are replaced by deuterons which is favorable for a good SNR in DMI.

The aims of this study were to demonstrate the feasibility of DMI to monitor [^2^H_9_] choline uptake in subcutaneous renal tumor models and to record the uptake and conversion of [6,6′-^2^H_2_]glucose simultaneously with that of [^2^H_9_]choline in these tumors.

## 2. Materials and Methods

### 2.1. Tumors and Animal Handling

Animal experiments were conducted according to institutional guidelines and regulations and were approved by the national Central Animal Experiments Committee (CCD) and the local animal welfare body (RU-DEC-2017-0038-12 and RU-DEC-2017-0038-013). Mice (age 6–8 weeks) were kept under specified pathogen free conditions and received food and water ad libitum. Human renal carcinoma cells were subcutaneously injected in the flank of athymic female BALB/c nu/nu mice (SKR17T-CA9-Luc cells) or of NSG (NOD SCID) mice (NU12 cells) and tumors were grown up to about 0.5 cm^3^ in size. Before the MR experiments, they were anesthetized by applying 1–2% isoflurane in a mixture of O_2_/N_2_O = 1:2 through a mask. Respiration rate was maintained at 60–80 bpm. Body temperature was monitored with a rectal probe and maintained at about 37 °C with heated air. A catheter was placed in a tail vein for the infusion of solutions with ^2^H compounds. About 30 min before the MR measurement, mice were IM injected with 12 µg atropine to prevent a possible cholinergic reaction of the choline bolus. At the time of the MR experiments, the animals weighed 24–38 g.

### 2.2. MR Hardware and IV Infusions

Mice were placed prone in the small bore magnet of an 11.7T BioSpec Avance III animal MR system (Bruker BioSpin GmbH, Ettlingen, Germany). The tumors were positioned inside a circular ^2^H coil with 16 mm diameter (76.8 MHz), which was surrounded by a circular ^1^H coil of 25 mm diameter (500.3 MHz).

Inside the magnet the mice were IV infused with a saline solution of ~0.2 mL in ~20 s containing either 0.05 g/kg [^2^H_9_]choline or 0.05 g/kg [^2^H_9_]choline combined with 1.3 g/kg [6,6′-^2^H_2_]glucose. The ^2^H compounds were obtained from Sigma-Aldrich (Zwijndrecht, The Netherlands).

### 2.3. Deuterium MR Spectroscopy and Imaging

First, T_2_ weighted RARE ^1^H MR images were recorded with the ^1^H coil to check the proper positioning of the tumors in the ^2^H coil (echo time (TE): 30 ms, repetition time (TR): 2500 ms, field of view (FOV): 20 × 20 mm, matrix 128 × 128 and a slice thickness (ST) of 1 mm) and T_1_ weighted 3D FLASH ^1^H MR images (TE: 2.3 ms; TR: 20 ms; flip angle: 10°, FOV: 33 × 33 × 33 mm; matrix 63 × 63 × 63) were acquired as reference images for the DMI. Guided by the anatomical images, a volume of interest (VOI) of variable size (e.g., 5 × 3 × 5 mm) was selected inside the tumor to optimize magnetic field homogeneity on the line width of the water signal using a PRESS sequence.

DMS was recorded with a pulse-acquire sequence using a 50 µs square excitation pulse of about 90° in the center of the coil. The pulse repetition time (TR) was 500 ms, the acquisition band width was 14 ppm and 300 averages were acquired in a total acquisition time of 2:24 min. 3D DMI was performed with a 1 ms composite RF pulse, optimized to provide circa five-fold immunity towards RF inhomogeneity over a spectral bandwidth >600 Hz, with TR = 400 ms and TE = 0.4 ms either at a high spatial resolution with a FOV of 30 × 30 × 30 mm and matrix size 15 × 15 × 15 (resulting in nominal voxels of 2 × 2 × 2 mm (8 µL) obtained in 37 min) or at low spatial resolution with a FOV of 33 × 33 × 33 and matrix size 9 × 9 × 9 (resulting in a nominal voxels of 3.7 × 3.7 × 3.7 mm (~50 µL) recorded in 2:24 min). The k-space data were acquired with spherical sampling in 3D.

### 2.4. Data Processing

Deuterium metabolic imaging data was handled in DMIWizard (Fourier transform, phasing, filtering, image reconstruction), a home-written Matlab-based graphical user interface for the processing of 1D DMS and 3D DMI data.

After FT of the time series of 45 FIDs, the ^2^H MR spectra were frequency aligned and, to improve the SNR, a moving average of 3 spectra was applied using jMRUI software [18]. Within jMRUI, the program AMARES was used to fit the signals of deuterated water (HOD), choline (Cho), glucose (Glc) and lactate (Lac), assuming a Lorentzian line shape. The glucose signal was approximated with two Lorentzian shaped lines, resonating 4.6 Hz apart. As additional prior knowledge in signal fitting, the relative resonance frequencies of the signals were included relative to HOD at 4.8 ppm (i.e. Glc1 at 3.83 ppm, Glc2 at 3.77 ppm, Cho at 3.2 ppm and Lac at 1.3 ppm) and line widths were constrained between 10 and 60 Hz.

Absolute tissue concentrations were determined using the initial natural abundance HOD signal as an internal reference assuming a natural abundance HOD concentration of 13.7 mM [8]. The tissue concentrations of compounds were normalized with respect to the number of deuterons attached to them.

### 2.5. Blood Samples

To determine ^2^H labeled compounds in blood, samples of 0.5 mL were obtained from a puncture of the heart of the mice after infusion. Plasma was derived from the blood samples by centrifugation for 5 min at 5000 rpm to separate it from cells. ^2^H NMR spectra of 200 µL plasma samples were recorded at 500 MHz on a Bruker AVANCE III vertical bore NMR system (Bruker BioSpin, Ettlingen, Germany) at a bandwidth of 12 ppm with 5 k data points and 512 acquisitions. The repetition time was 5 s. If needed measurements per time point were frequency aligned and averaged in jMRUI and for fitting AMARES was applied using the same prior knowledge as for the in vivo spectra.

## 3. Results

### DMS and DMI of Renal Tumor after [^2^H_9_]choline Infusion

**Figure 1:**
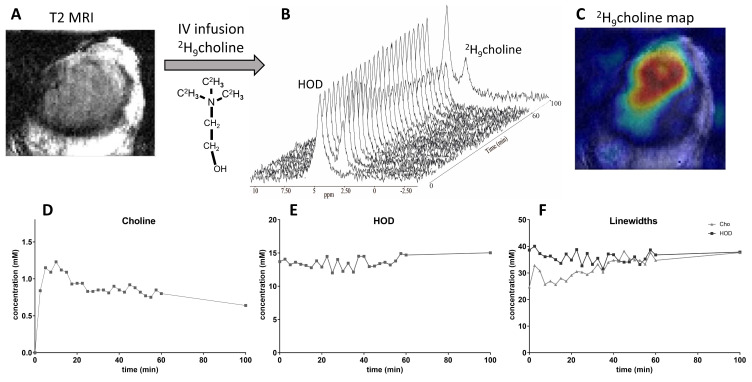
Deuterium MR of a human renal tumor (SKRC17T) subcutaneously grown in a BALB/c mouse. (**A**). T2 MRI of the tumor. The chemical structure of [^2^H_9_]choline is also indicated. (**B**). Unlocalized ^2^H MR spectra obtained with a surface coil at a time resolution of 2:30 min. The first spectrum recorded before the infusion of choline only shows a natural abundance HOD signal at 4.8 ppm. Immediately after the infusion of a bolus of about 0.2 mL with [^2^H_9_]choline, a signal for this compound is observed at about 3.2 ppm. Within ~5 min after the infusion this signal has reached its maximum value and then slowly declines over time. (**C**). A metabolic map of [^2^H_9_]choline reconstructed from a high resolution DMI obtained between 60 and 100 min post-infusion. (**D**). [^2^H_9_]choline tissue concentration (mM) as a function of time post-infusion. After a rapid initial increase, it slowly decreases over time. (**E**). HOD tissue concentration (mM) as a function of time. Over the measurement period of 100 min the HOD concentration essentially remains constant. (**F**). Fitted linewidths (Hz) of the [^2^H_9_]choline and HOD resonances as a function of time. That of HOD essentially remains constant, while that of [^2^H_9_]choline increases.

To test if we could monitor the uptake of ^2^H_9_ choline in a subcutaneous tumor, we infused 0.05 g/kg of a saline solution of about 0.15 mL of this compound for ~15 seconds in the tail vein of mice carrying an SKRC17T tumor. On a T2 weighted MR image recorded with the ^1^H coil, the tumor can be seen (Figure 1A). In a volume of 7 × 6 × 7 mm, the magnetic field homogeneity in the tumor was optimized to a water line width of 65 Hz. The ^2^H MR spectrum recorded with the ^2^H coil, without further localization, before the infusion only showed a signal for natural abundance HOD (Figure 1B). Immediately after the infusion a ^2^H signal appeared at ~3.2 ppm, well separated from that of HOD, which is assigned to the deuterated methyl groups of choline compounds. From its signal integral we deduced that its tissue concentration increased to a maximum level of ~1.2 mM [^2^H_9_]choline at ~5 min post-infusion and then declined to 0.9 mM at ~15 min post-infusion, after which it slowly decreased to ~0.7 mM at 100 min post-infusion (Figure 1D). Over this time period, the HOD signal remained almost constant (Figure 1E) and no other resolved ^2^H signals were observed. Shortly after the infusion, the unfiltered line width of the unlocalized signal was about 38 Hz for HOD and was about 25 Hz for [^2^H_9_]choline. In the 100 min thereafter, the linewidth of HOD did not change, but that of [^2^H_9_]choline steadily increased to about 36 Hz (Figure 1F). From a high resolution 3D DMI obtained between about 60 and 90 min post-infusion, a [^2^H_9_]choline map could be reconstructed (Figure 1C).

**Figure 2:**
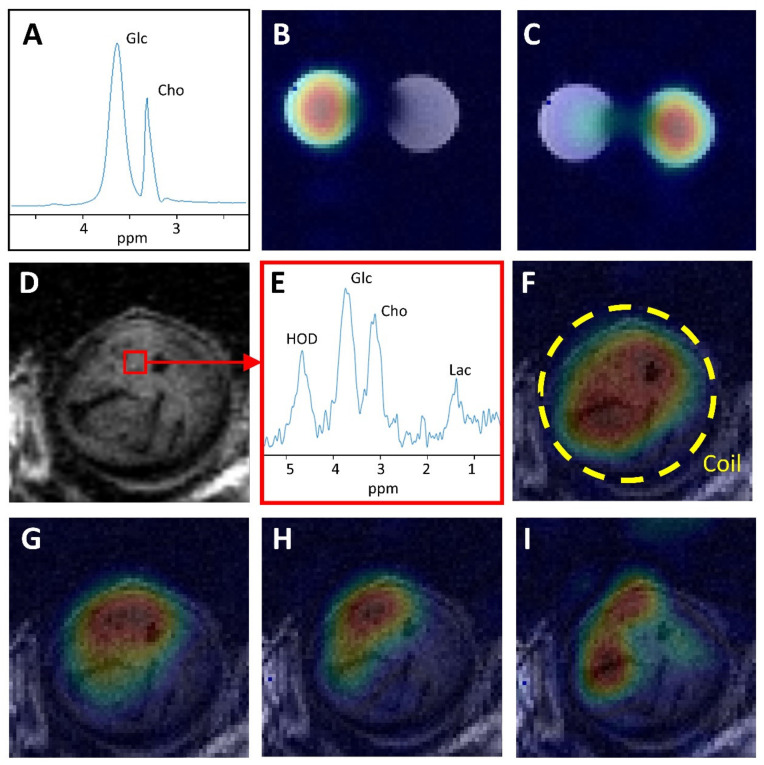
Deuterium metabolic imaging study of deuterated glucose and choline in phantom and in a human renal tumor subcutaneously growing in mice. (**A**). An unlocalized ^2^H MR spectrum of two tubes, one filled with a saline solution of ^2^H_9_ choline (63 mM) and the other with a saline solution of [6,6′-^2^H_2_]glucose (0.71 M). The glucose signal is completely separated from the choline signal. (**B**). [6.6′-^2^H_2_]glucose map reconstructed from high resolution DMI of both tubes. (**C**). [^2^H_9_]choline map reconstructed from high resolution DMI of both tubes. (**D**). T2 weighted MR image of a slice through a NU12 renal carcinoma growing in the flank of an NSG mouse. A voxel of 8 µL is indicated, which was selected from a high resolution 3D DMI recorded in 37 min after IV application of a mixture of [6,6′-^2^H_2_]glucose and [^2^H_9_]choline. (**E**). The ^2^H MR spectrum of the 8 µL voxel selected from the tumor shows signals for water (HOD), glucose (Glc), choline (Cho) and lactate (Lac). The spectrum was filtered with a 2Hz exponential line broadening. The glucose and choline signals are clearly separated. (**F**). HOD map reconstructed from 3D DMI. (**G**). Glucose map reconstructed from 3D DMI. (**H**). Choline map reconstructed from 3D DMI. (**I**). Lactate map reconstructed from 3D DMI. The maps are oriented parallel to the ^2^H RF coil (indicated by a circle in F) and projected on top of T1 MR images. The metabolic color maps were scaled to the highest intensity of the corresponding metabolite signal.

To test how well the ^2^H signals of [^2^H^9^]choline and of [6,6′-^2^H_2_]glucose are separated we recorded ^2^H MR spectra of a phantom containing two tubes, one filled with [^2^H_9_]choline and the other filled with [6,6′-^2^H_2_]glucose. An unlocalized ^2^H MR spectrum of the phantom showed that the ^2^H signals of choline and glucose are well-resolved (Figure 2A). Using a single Lorentzian line to fit these resonances resulted in a line width of 17.1 ± 4.2.Hz (SD of fit error) for choline and 32.2 ± 4.3 Hz for glucose. The measurement of a 3D high resolution DMI of the phantom enabled the reconstruction of separate maps for glucose (Figure 2B) and choline (Figure 2C) in the tubes.

Subsequently, we investigated whether it was also possible to observe separated choline and glucose signals in ^2^H spectra of a subcutaneous tumor by performing a 20 s infusion of a solution with [^2^H_9_]choline and [6,6′-^2^H_2_]glucose in the tail vein of an NSG mouse carrying a NU12-Luc-GFP tumor (Figure 2D) and recording a high resolution 3D DMI. In a ^2^H MR spectrum of an 8 µL voxel in the DMI of the tumor, the deuterated choline and glucose signals are well separated, and a resolved signal for deuterated lactate is also observed (Figure 2E). The tissue concentration of [^2^H_9_]choline (averaged over the time of the DMI recording of 37 min) was about 2 mM and its line width was 19 Hz. For each of the deuterated compounds with signals in the ^2^H MR spectra, we reconstructed metabolic maps parallel to the circular RF coil: HOD (Figure 2F), glucose (Figure 2G), choline (Figure 2H), and lactate (Figure 2I). Although signal distribution in these maps is also determined by the sensitive field of the RF coil, a comparison of the signal intensities in the maps of the metabolites (which is expected to be the same if only determined by the RF field) indicates differences in their distributions.

**Figure 3:**
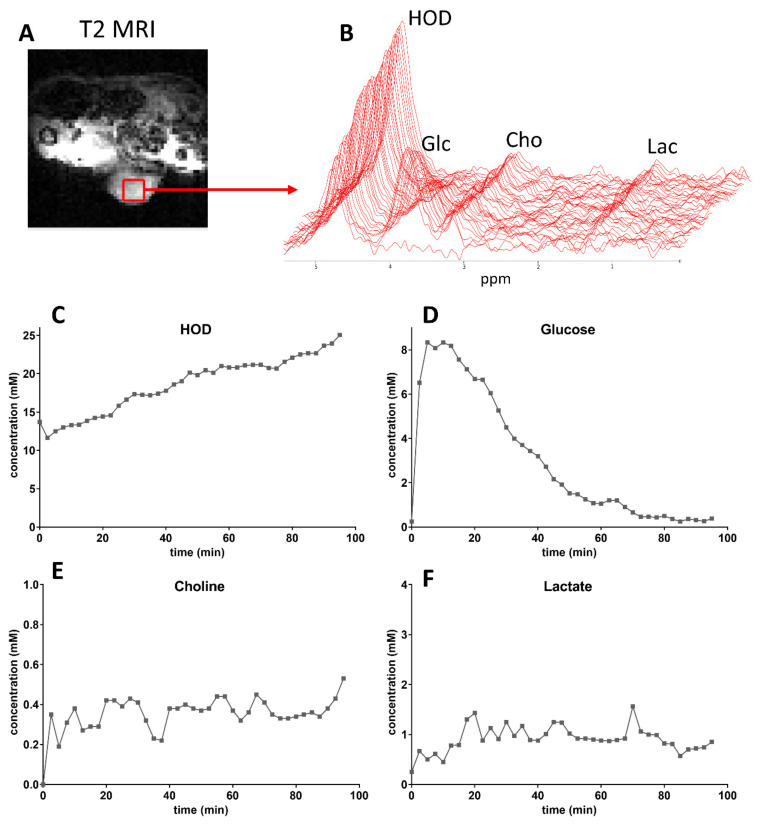
Low spatial resolution 3D DMI (50 µL voxels), recorded at a time resolution of 2:24 min, from an SKRC 17T-CA9-Luc tumor growing in the flank of a mouse. (**A**). T2 weighted MRI with a selected voxel inside the tumor indicated by a red box. (**B**). Time series of ^2^H MR spectra consecutively recorded from this voxel over a period of 90 min showing an increasing HOD signal, an increasing and decreasing glucose signal (**C**,**D**), a rapidly increasing and then constant choline signal and a signal for lactate appearing during the decline of the glucose signal. (**E**,**F**). Time curves of metabolite tissue concentrations (mM).

To examine if we can follow the conversion of [6,6′-^2^H_2_]glucose into lactate over time in the presence of [^2^H_9_]choline in a tumor, we recorded sequential low spatial resolution DMIs at a time resolution of 2:24 min after the infusion of a mixture of these compounds into mice carrying a subcutaneous SKRC 17-CA9-Luc tumor (Figure 3A). A stack plot of ^2^H MR spectra and the calculated tissue concentrations of compounds in these spectra as a function of time are displayed in Figure 3B–F. The HOD level is steadily increasing over time (Figure 3C), while the glucose content is increasing to a maximum concentration of about 8 mM in 5–10 min and then declines (Figure 3D). During this decline, a signal at 1.3 ppm for lactate appeared (Figure 3E). The [^2^H_9_]choline tissue concentration increased to values between 0.3 and 0.4 mM and remained stable up to 100 min. The line width of the [^2^H_9_]choline signal increased from about 10 Hz immediately after infusion to about 15 Hz after 100 min.

Similar results were obtained in two other mice carrying tumors of the same batch. For all 3 tumors the average tumor tissue concentration between 20 and 60 min post-infusion was 0.32 ± 0.05 mM for [^2^H_9_]choline was 0.32 ± 0.05 mM and was 0.86 ± 0.27 mM for deuterated lactate. The maximum average [6,6′-^2^H_2_]glucose tissue concentration at 10 min post-infusion was 8.87 ± 2.58 mM. Initial linewidths of compound signals in the spectra of all tumor DMIs, recorded shortly after the infusion, depended on the magnetic field shimming results.

**Figure 4:**
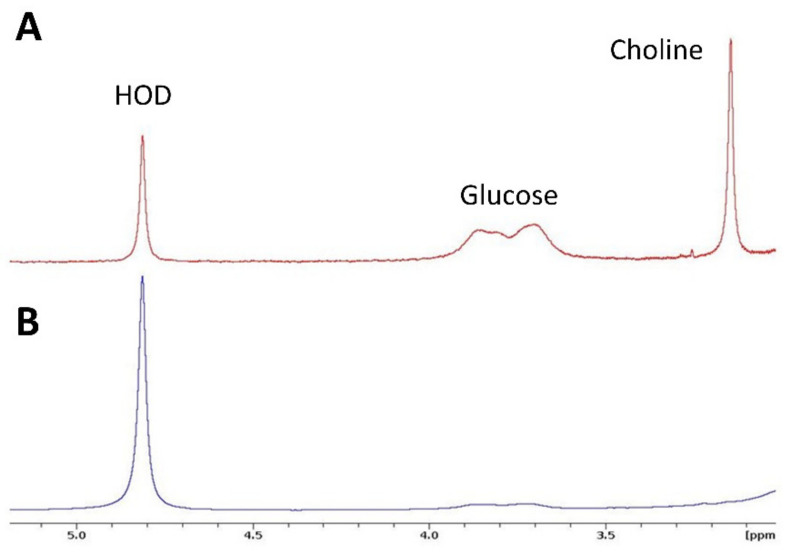
^2^H MR spectra recorded from plasma samples after infusion of a mixture of [6,6′-^2^H_2_]glucose and [^2^H_9_]choline in mice carrying a subcutaneous renal tumor. (**A**). Spectrum taken immediately after infusion. (**B**). Spectrum taken 15 min after infusion. The ^2^H signal for glucose is strongly reduced and that for choline signal is absent. Vertical scales are arbitrary.

In ^2^H NMR spectra of blood plasma taken immediately after infusion of a mixture of [6,6′-^2^H_2_]glucose and [^2^H_9_]choline, signals of both compounds can be detected next to that of HOD (Figure 4A). Their concentrations were estimated to be 2.6 mM for choline and 11.6 mM for glucose. From spectra taken at 15 min or longer after infusion, no choline signal could be detected and the glucose signal was strongly decreased (Figure 4B). No significant signal for [^2^H_9_]betaine (the first breakdown product of choline at ~3.4 ppm) was detected.

## 4. Discussion

In this study, we developed a protocol to follow the uptake and to image the presence of [^2^H_9_]choline in renal tumors subcutaneously growing in the flank of mice via ^2^H MR spectroscopy (DMS) and imaging (DMI) after an IV bolus administration of this compound. In addition, we demonstrated that DMI can monitor the uptake of [^2^H_9_]choline in these tumors simultaneously with that of [6,6′-^2^H_2_]glucose, and its conversion into lactate after bolus administration of a mixture of both compounds. In ^2^H NMR spectra obtained of blood plasma at 15 min post-infusion no [^2^H_9_]choline signal was present, in agreement with the half-life of plasma choline of less than 1 min in mice [19]. This implies that the [^2^H_9_]choline signal seen in the in vivo ^2^H MR spectra arises from tumor tissue.

Previously, the uptake of [^2^H_9_]choline has been monitored in an MCF7 human breast tumor, growing subcutaneously in CD1 mice, via ^2^H MR spectroscopy at 4.7T of a single voxel of 1 cm^3^ during continuous low dose infusion (16 µmol/kg/min) [20]. In contrast to our study, a [^2^H_9_]choline signal only appeared ~40 min after the start of infusion, but then increased to a tissue concentration of ~1.5 mM, which is comparable to the concentrations observed in our renal subcutaneous tumors (0.3–2 mM). During infusion, the plasma level of [^2^H_9_]choline in CD1 mice reached a level of 222 ± 22 µM, while it was estimated in our mice to be 2.6 mM immediately after the bolus application. The higher field strength contributed to a better spectral resolution in our study, but also the smaller selected volumes of 50 µL and 8 µL and possibly the tissue composition of the tumor favored an improved field homogeneity and spectral resolution. Recently, the specific accumulation of [^2^H_9_]choline in a tumor in the rat brain by DMI has been demonstrated [21].

In our experiments with only [^2^H_9_]choline infusion, the HOD signal did not change much during the measurement time up to about 90 min, which is expected with a small loss of deuterons from [^2^H_9_]choline or its downstream products in processes involving exchange with water. With this protocol of bolus administration, we also did not detect any signal for [^2^H_9_]betaine, the main breakdown product of choline compounds [22].

In all experiments with [^2^H_9_]choline infusion, its signal in tumors rapidly increased to a maximum level within 5 min and then slowly decreased or remained stable up to 100 min post-infusion. As we cannot discriminate among the choline species that contribute to the [^2^H_9_]choline signal, it may be that the composition of these species change over time. Recent ^2^H NMR studies of biopsies from brain tumors in the rat supplied with [^2^H_9_]choline indicate that choline is metabolized into phosphocholine and glycerophosphocholine within the tumor, although substantial amounts of free choline still seem to be present after 36 min [23]. In this respect, it is interesting that in our experiments the line width of the [^2^H_9_]choline signal increased over time, apparently because of the contribution of the signals of other choline compounds with a slightly different chemical shift.

Because the IV application of a 0.05 g/kg [^2^H_9_]choline bolus may give an acute cholinergic reaction [24], we applied atropine to the mice as preventive measure. To avoid this application, the amount of [^2^H_9_]choline can be decreased and/or the infusion time can be extended. In other supplementation studies (particularly in humans), choline is often administered as a continuous infusion or orally [21,25,26].

Choline imaging with enhanced sensitivity has also been explored with hyperpolarization by dynamic nuclear polarization (DNP) of [1,1,2,2 ^2^H_4_, 1-^13^C] choline [27] and the in vivo detection of DNP hyperpolarized ^15^N choline in the rat was demonstrated [28]. In these experiments, precautions also had to be taken to avoid cholinergic reactions because a bolus infusion with relatively high choline concentrations (~90 mM) was required. The intrinsic sensitivity of MR experiments using hyperpolarization is much better than that of ^2^H MR experiments. Although no one-to-one comparisons have been made yet, we anticipate that this difference will be largely compensated by the much longer time available for data acquisition in ^2^H MR experiments and the nine deuterons contributing to the [^2^H_9_]choline signal. Apart from this sensitivity issue, ^2^H MR experiments are less complicated to perform and easier to analyze than hyperpolarization MR experiments.

Increased steady state levels of choline compounds are a common finding in tumors and are associated with demands for enhanced phospholipid metabolism to support cellular membrane synthesis in oncogenesis and tumor progression. This property has been explored in ^1^H MR spectroscopic imaging by using the (relative) intensity of the overlapping signals of all choline compounds together as a non-invasive diagnostic marker for several cancers such as in the brain, prostate and breast [12]. An increased total choline signal may serve to localize the tumor and its quantified intensity to determine tumor stage or aggressiveness [15] or to evaluate the effect of tumor treatments [29,30]. Choline tracer imaging (such as in ^11^C choline PET or in [^2^H_9_]choline DMI) provides a more dynamic view on choline uptake than the steady state levels of MRS detectable choline metabolites. Although ^1^H MRSI offers a better spectral resolution than DMI, it faces the technical challenge that the total choline signal has to be separated from background proton signals, in particular of water and lipids. An even better spectral resolution is provided by ^13^C MRS, but at the cost of a lower sensitivity, unless DNP methods are applied (vide supra).

In this feasibility study we noticed that tumors may accumulate different levels of [^2^H_9_]choline and with variable spatial distributions. In future studies this has to be correlated with molecular features of these tumors such as proliferation markers, glucose and choline transporters, and with tumor heterogeneity. In addition, the value of our DMI approaches should be tested in tumors of different origin and body location.

The simultaneous monitoring by DMI of the uptake of choline and glucose and its conversion over time, after combined [6,6′-^2^H_2_]glucose and [^2^H_9_]choline infusion, was demonstrated for renal SKRC 17-CA9-Luc tumors. The concentration-time curve of [6,6′-^2^H_2_]glucose recorded for these tumors are comparable to that in subcutaneously growing EL4 tumors recorded under similar infusion and DMI conditions, taking normalization with initial [HOD] into account [11]. However, the deuterated lactate concentration in our tumors was much lower, which indicates more oxidative metabolism or rapid lactate processing. As no metabolites related to the TCA cycle have been detected (e.g., glutamate), it is likely that lactate processing or removal is more efficient in these renal tumors.

To apply DMI of choline and glucose, separately or combined, to humans at lower field strengths (≤7T), it is necessary that their signals are resolved from HOD and from each other. The line width of HOD from DMI volumes in the tumor (15–25 Hz) was larger than that reported for the human brain at 4 and 7T [31], which is likely due to more difficult magnetic field shimming conditions. For choline, we found line widths of about 10 to 20 Hz. As the spectral separation between HOD and choline signals is ~1.5 ppm, which translates to ~30 Hz difference at 3T, and assuming no field dependent change in line width [31], it follows that these signals can also be separately observed at this clinical field strength. Likewise, we anticipate that glucose and choline signals (~0.5 ppm apart) can be distinguished at 7T, but at 3T this may more critically depend on shimming results. However, the glucose signal disappears within ~60 min post-infusion, which would allow the observation of the choline signal as its intensity then has not changed much yet.

FDG PET is widely employed to detect tumors in the human body and ^11^C or ^18^F choline PET is applied in more specific cases (such as in the characterization of prostate and other cancers in the body [14,32,33]). Because of the high sensitivity of PET, isotope labeled compounds can be applied at trace amounts, but when endogenous compounds occurring at high body concentration are involved (e.g., glucose and choline), DMI may be considered as a non-radioactive alternative. This has several additional advantages, such as that these compounds can be monitored simultaneously, that the metabolism of glucose to lactate and other compounds and to HOD can be evaluated and that it can be combined with multi-parametric MRI. The application of other combinations of deuterated compounds in tumor studies are foreseeable, as long as their signals are spectrally resolved, for instance choline with the conversion of fumarate into malate to assess tumor cell death after treatments [34] or an inert deuterated compound to assess tumor perfusion.

DMI does require that MR machines are equipped with dedicated ^2^H RF coils. For humans, RF coils of sufficient quality for DMI at 7T to diagnose tumors in the brain have been presented that appear to offer a spatial resolution comparable to that of FDG PET [9]. Recently, dedicated human ^2^H body coils were also shown to be able to record DMIs, such as those of the liver after [6,6′-^2^H_2_]glucose application [9,35]. However, it remains to be seen what spatial resolution and tissue coverage can be achieved to detect and characterize tumors in the body as compared to FDG and choline PET, which essentially provide whole body imaging. The SNR of DMI experiments can be improved by signal denoising, as recently demonstrated for DMI at 3T [36], and also by more optimal MR detection methods [37], which can be employed for better spatial resolution or faster imaging.

## 5. Conclusions

In this study, we demonstrate that DMI of [^2^H_9_]choline uptake in tumors subcutaneously growing in the mice is feasible. Moreover, we show that the simultaneous monitoring of [^2^H_9_]choline and [6,6′-^2^H_2_]glucose and its catabolic product lactate in tumors via DMI is possible. This enables researchers to obtain information from two main tumor properties at the same time, enhanced glucose uptake and catabolism and choline uptake, and therefore has the potential to provide a more specific metabolic characterization of tumor lesions than that obtained with single tracer, single parameter metabolic imaging.

As DMI of glucose uptake and its metabolic conversions is feasible in patients, the simultaneous performance of choline and glucose DMI has clinical potential.

## Data Availability

Data of this study are available by the last author upon reasonable request.
